# Co-expression of cancer stem cell markers, SALL4/ALDH1A1, is associated with tumor aggressiveness and poor survival in patients with serous ovarian carcinoma

**DOI:** 10.1186/s13048-021-00921-x

**Published:** 2022-01-28

**Authors:** Mina Sharbatoghli, Parisa Shamshiripour, Fahimeh Fattahi, Elham Kalantari, Zohre Habibi Shams, Mahshid Panahi, Mehdi Totonchi, Zeynab Asadi-Lari, Zahra Madjd, Leili Saeednejad Zanjani

**Affiliations:** 1 Oncopathology Research Center, Iran University of Medical Sciences (IUMS), Tehran, Iran; 2 Department of Pathology, Faculty of Medicine, Iran University of Medical Sciences, Tehran, Iran; 3 Department of Molecular Medicine, Faculty of Advanced Technologies in Medicine, Iran University of Medical Sciences, Tehran, Iran; 4 Department of Stem Cells and Developmental Biology, Cell Science Research Center, Royan Institute for Stem Cell Biology and Technology, ACECR, Tehran, Iran; 5 Department of Genetics, Reproductive Biomedicine Research Center, Royan Institute for Reproductive Biomedicine, ACECR, Tehran, Iran; 6 Department of Biology, Faculty of Science, University of Toronto, Toronto, Canada

**Keywords:** Serous ovarian carcinoma (SOC), SALL4, ALDH1A1, Prognosis, Immunohistochemistry (IHC), Tissue microarray (TMA)

## Abstract

**Background:**

Spalt-like transcription factor 4 (SALL4) and aldehyde dehydrogenase1 family member A1 (ALDH1A1) expressing cells have been characterized as possessing stem cell-like properties known as cancer stem cell marker in serous ovarian carcinoma (SOC).

**Methods:**

The association between SALL4 and ALDH1A1 was observed based on literature review and bioinformatics tools. Therefore, this study aimed to investigate the association between the co-expression of SALL4/ALDH1A1 proteins and clinicopathological parameters and their prognostic value in SOC patients using immunohistochemical staining on tissue microarrays (TMAs). Furthermore, benign tumors and normal tissue samples were compared with the expression of the tumor tissue samples.

**Results:**

Increased co-expression of SALL4/ALDH1A1 was found to be significantly associated with the advanced FIGO stage (*P* = 0.047), and distant metastasis (*P* = 0.028). The results of Kaplan–Meier survival analysis indicated significant differences between disease- specific survival (DSS; *P* = 0.034) or progression-free survival (PFS; *P* = 0.018) and the patients with high and low co-expression of SALL4/ALDH1A1, respectively. Furthermore, high level co-expression of SALL4/ALDH1A1 was a significant predictor of worse DSS and PFS in the univariate analysis. The data also indicated that the co-expression of SALL4/ALDH1A1 was an independent prognostic factor affecting PFS. Moreover, the co-expression of SALL4/ALDH1A1 added prognostic values of DSS in patients with SOC who had grade III versus grade I in multivariate analysis.

**Conclusions:**

Our data demonstrated that high co-expression of SALL4/ALDH1A1 was found to be significantly associated with tumor aggressiveness and worse DSS or PFS in SOC patients. Therefore, co-expression of SALL4/ALDH1A1 may serve as a potential prognostic biomarker of cancer progression in these cases.

**Supplementary Information:**

The online version contains supplementary material available at 10.1186/s13048-021-00921-x.

## Background

Ovarian cancer (OC) is the eighth common gynecologic malignancy and is the fifth cause of cancer-related death among women. The American Cancer Society estimates about 21,410 new cases of OC and the mortality rate of 13,770 women of OC in 2021 [1].

Epithelial ovarian carcinoma (EOC) is the most common OC, accounting for 95% of ovarian malignancies, that comprises diverse histological subtypes as follows: serous, mucinous, clear-cell, endometrioid, transitional cell or brenner tumor, and mixed epithelial carcinoma [2]. Specifically, EOC as a serous ovarian carcinoma (SOC), is diagnosed at advanced stages of disease in 70% of cases because of non-specific sign and symptoms of ovarian tumors [3]. For these patients, surgery followed by chemotherapy remains the standard of care [4]. Moreover, there are some common serum tumor FDA approved biomarkers for screening high-risk OC women, including carcinoembryonic antigen (CEA), cancer antigen 125 (CA125), human epididymis protein 4 (HE4), risk of ovarian malignancy algorithm (ROMA), ova1, and overa [5]. Notably, these biomarkers are not applied as a prognostic biomarker and they have low sensitivity and low accuracy [6]. Hence, an optimal biomarker with high accuracy is essentially needed that improves the prognostic biomarker.

Over the years, many studies have demonstrated and confirmed tumor initiation, progression, recurrence, and drug resistance in various types of cancer because of a subpopulation of tumor cells called cancer stem cells (CSCs). These cells exhibited a variety of characteristics similar to normal stem cells (NSCs) or progenitor cells, including self-renewal, multipotency, and differentiation into a range of cell types that led to tumor growth and heterogeneity [7, 8]. Various markers are used for the detection of these subpopulation of tumor cells with stem cell properties. However, there exist common characteristics between the CSC markers reported in various tumor types as the investigation and selection of the biomarkers depend to a large extent on the tissues [9, 10]. Moreover, the results achieved from the investigation of relative expression levels of the panel of CSC markers in in-vitro and population studies indicated relationships between the expression of some CSC markers [11, 12] while there is no correlation between others [10].

Our review of the literature regarding CSC markers indicated that spalt-like transcription factor 4 (SALL4) and Aldehyde dehydrogenase 1 (ALDH1) are two biomarkers of NSCs and ovarian CSCs [13, 14]. Furthermore, the expression of SALL4 can be related to the expression levels of other CSC markers such as ALDH1 [15, 16]. SALL4 is a zinc finger transcription factor expressed in embryonic stem cells, and plays an important role in the regulation of development, embryogenesis, organogenesis, and preservation of self-renewal and pluripotency [17]. It has been observed that the expression of SALL4 is silenced in entirely differentiated cells while its expression is reactivated in various types of cancer such as central nervous system tumor [18], lung cancer [19], colorectal cancer (CRC) [20], liver cancer [21], leukemia [22], endometrial cancer [23], breast cancer (BC) [24], glioma [25], and gastric cancer [26]. Previous studies have shown that SALL4 were highly expressed in SOC tissues in the levels of mRNA and protein in OC cell lines, and germ cell tumors [14, 27].

Studies in cancer also indicated that overexpression of SALL4 can increase the proliferation, migration, and invasion of cancer cells through targeting epithelial mesenchymal transition (EMT) [26, 28, 29]. Moreover, high expression of SALL4 is related to low survival and has been noticed as a prognostic factor in the patients with BC and gliomas [24, 25].

Another CSC marker, ALDH1 is a detoxifying cytosolic enzyme, responsible for the oxidation of intracellular aldehydes that are critical for early differentiation, self-renewal of stem cell and CSC subpopulation regulation [30]. ALDH1A1 is a member of the 19 human ALDH subfamilies whose expression is correlated with human CSC and chemotherapy resistant [31]. Recently a meta-analysis study demonstrated that higher expression of ALDH1 is a prognostic biomarker for head and neck [32], BC [33], CRC [34], and lung cancer [35]. In OC, ALDH1A1 was found to be associated with stemness and poor prognosis [36]. The differential expression pattern of ALDH1A1 in OC tissue is also reported. Penumatsa et al. observed that ALDH1A1 expression is significantly reduced in malignant ovarian tumor while it is relatively unchanged in benign tumors in comparison to normal ovary [37]. Whoever Muhammad et al. indicated that mucinous adenocarcinoma in grade II expresses a high level of ALDH1A1 protein in comparison with borderline mucinous and benign serous tumors [38].

In the present study, at primary search and literature review, alterations in mRNA levels of SALL4 and ALDH1 as biomarkers in SOC patients were comprehensively analyzed via Gene Expression Profiling Interactive Analysis 2 (GEPIA2) databases. Additionally, the relationship of SALL4 and ALDH1 with other ovarian CSC markers were investigated by protein-protein interaction (PPI) network analysis and enrichment analysis. Then, for the first time, the co-expression of SALL4 and ALDH1A1 proteins as a prognostic biomarker was evaluated in a series of formalin-fixed paraffin-embedded (FFPE) tissues for SOC using the immunohistochemistry (IHC) technique on tissue microarray (TMA) slides. The association between co-expression levels of SALL4 and ALDH1A1 proteins with clinicopathological features as well as clinical outcomes was investigated.

## Materials and methods

### Investigation of the expression of SALL4 and ALDH1A1 using bioinformatics tools

The GEPIA2 database was used to compare mRNA expression of SALL4 and ALDH1A1 between tumors and normal tissues. The GEPIA2 online tool is a valuable resource that allows users to perform all expression analyses at the isoform level according to different tumor and normal samples from the TCGA and the GTEx databases [39]. Moreover, to find interactions and relationships between SALL4 and ALDH1A1 markers together and with other CSCs markers in OC, CSCs markers were tracked in earlier studies. Consequently, 83 protein markers as CSC markers in OC were extracted (Supplementary Table 1). Then, PPI networks were constructed for these genes by stringApp)confidence score ≥ 0.15 ([40] in Cytoscape software [41]. The relationships of SALL4 and ALDH1A1 were evaluated by the GeneMANIA-based webserver. As a general-purpose network search engine, this tool indicates interactions of genes and predicts gene function [42]. In this study, in order to exhibit features of SALL4 and ALDH1A1, biological process (BP) and molecular function (MF) as well as the pathways with which these two genes were involved were obtained through enrichment analysis by ClueGO plug-in [43] using Cytoscape software. Pathway enrichment analysis was carried out according to KEGG [44], Reactome [45], and WikiPathways [46] databases.

### Patients and sample collection

A total of 45 FFPE tissue blocks from SOC tumors were collected from the Firozgar Hospital in Tehran, Iran, during 2011–2018. None of the patients had a history of chemotherapy or radiation therapy. Clinicopathological parameters, including tumor size (maximum tumor diameter), age, Federation International of Gynecology and Obstetrics (FIGO) stage, histological grade, vascular invasion (VI), lymph node (LN) metastasis, fallopian tube involvement, omentum involvement, cervix involvement, myometrium involvement, endometrium involvement, vagina involvement, peritoneum involvement, lymphovascular space invasion, perineural invasion (PI), colon involvement, small intestine involvement, post-cul-de-sac involvement, paracolic lymph node involvement, distant metastasis, and tumor recurrence were obtained using hematoxylin and eosin (H&E) stained slides and the medical information. In addition, 37 benign tumors of SALL4 and 15 benign tumors of ALDH1A1 as well as 20 normal tissue samples were collected in this survey to compare the expression levels of SALL4 and ADLH1A1 across a wide range of tissue samples.

Disease-specific survival (DSS) was described from the time of ovariectomy up to the date of death caused by cancer. Progression-free survival (PFS) was explained as the time interval between the first surgery and the last follow-up visit when the patient did not show any detectable disease, recurrence, or metastasis. The stage and grade were considered with reference to the FIGO -cancer report 2018 classification and College of American Pathologist (CAP), 2018, for OC [47, 48].

### Tissue microarray (TMA) construction and immunohistochemistry protocol

H&E stained slides were reviewed by a pathologist (M.P) and the most representative areas of tumors was marked on the slides. Selected areas were punched out from each donor block using tissue microarray instrument (Minicore; ALPHELYS, Plaisir, France) and assembled into a recipient TMA blocks [49]. The TMA blocks were constructed in three copies, each containing one sample from a different region of the tumor [50]. Afterwards, the TMA blocks were sectioned for further immune staining [51, 52]. TMA slides were deparaffinized in 60 °C and rehydrated in xylene and serial dilutions of alcohol; 100, 96, and 70%; respectively for 5 min. For blocking endogenous peroxidase activity, slides were co-incubated with 3% H2O2 for 20 min at 25 ° C.

Antigen retrieval were carried out by autoclaving the sections in citrate buffer (10 mM, pH 6.0) at 95-100 °C for 11 min. After conducting three wash steps with tris buffer saline (TBS, pH: 7.4), slides were incubated with protein blocker (Dako, CA, USA) for 15 min. Afterwards, TMA slides were incubated overnight with anti-SALL4 antibody (Gifted by Avicenna Research Institute, Monoclonal Antibody Research Center (MARC)) and ALDHA1 (Ab52492, Abcam, Cambridge, MA, USA) with 1/1000 and 1/200 (optimal dilutions), respectively as the primary antibodies at 4 °C. After three wash steps, slides were incubated with secondary antibody (anti-mouse-rabbit HRP polymer (EUROMAB, UMR1000PD, USA)) for 40 min. Visualization of immune signals were done by 3, 3′-diaminobenzidine (DAB, Dako, Glostrup, Denmark) substrate as chromogen for 5 min at 25 ° C. Afterwards hematoxylin was added to counterstain the slides. Dehydration steps were conducted using Xylene and serial dilutions of alchohol 70, 96, and 100%). Slides were imaged using light microscope. In this study, human testis seminoma tissues and human normal liver tissues were used as a positive control for anti-SALL4 and anti-ALDH1A1 antibody, respectively. While Tris-buffered saline (TBS), instead of the primary antibody, was used as a negative control to validate the nonspecific bindings of secondary antibody. In addition, rabbit immunoglobulin G (Invitrogen, Thermo Fisher Scientific, Waltham, MA, USA) was used as isotype control to confirm the nonspecific bindings of the primary antibody.

### Scoring system of IHC slides

TMA tissue sections were scored by two pathologists (M.P. & M.S.) blinded to clinicopathological features semi-quantitatively. Scoring evaluation was carried out with reinvestigation of the overall distribution of the tumor cells at 10× magnification. Positive cells were then assessed, semi-quantitatively, at higher magnifications (20× or 40×). The intensity of the mentioned markers staining was scored as 0 (absent), 1 (weak), 2 (moderate) or 3 (strong). Positive cells percentage were valued semi-quantitatively with a score ranging from 1 to 100%. The overall score was obtained by Histochemical score (H-score) for each case by multiplying the intensity of staining by the percentage of positive cells and a finals score of 0 to 300 was given to each core. TMA blocks were constructed in three copies of the most representative area of each tumor and final score of each tumor was given following an agreement between scorers of three replicates. The mean of H-scores of SALL4 and ALDH1A1 (H-score = 60, 135, respectively) were used as a cutoff point to categorize the tumors as with high or low expressions.

### Statistical analysis

The statistical analyses were performed by SPSS 22.0 (IBM Corp, USA) and the categorical data were described by N (%), valid percent, and quantitative data, including mean (SD) and median (Q1, Q3). For pairwise comparison between tumor tissues, benign tumors as well as normal tissue samples Kruskal–Wallis and Mann–Whitney *U* tests were done. The association between the co-expression of SALL4/ALDH1A1 proteins and clinicopathological features was applied using Pearson’s chi-square test. Furthermore, Kaplan–Meier method was employed to draw DSS and PFS curves, and the log-rank test was performed to compare the estimated curves between the groups with 95% confidence intervals (CI). The Cox proportional hazards regression model was adopted to perform univariate and multivariate analyses. Also, *P* < 0.05 was considered statistically significant.

## Results

### Bioinformatics approaches about SALL4 and ALDH1A1 in SOC

**Fig. 1:**
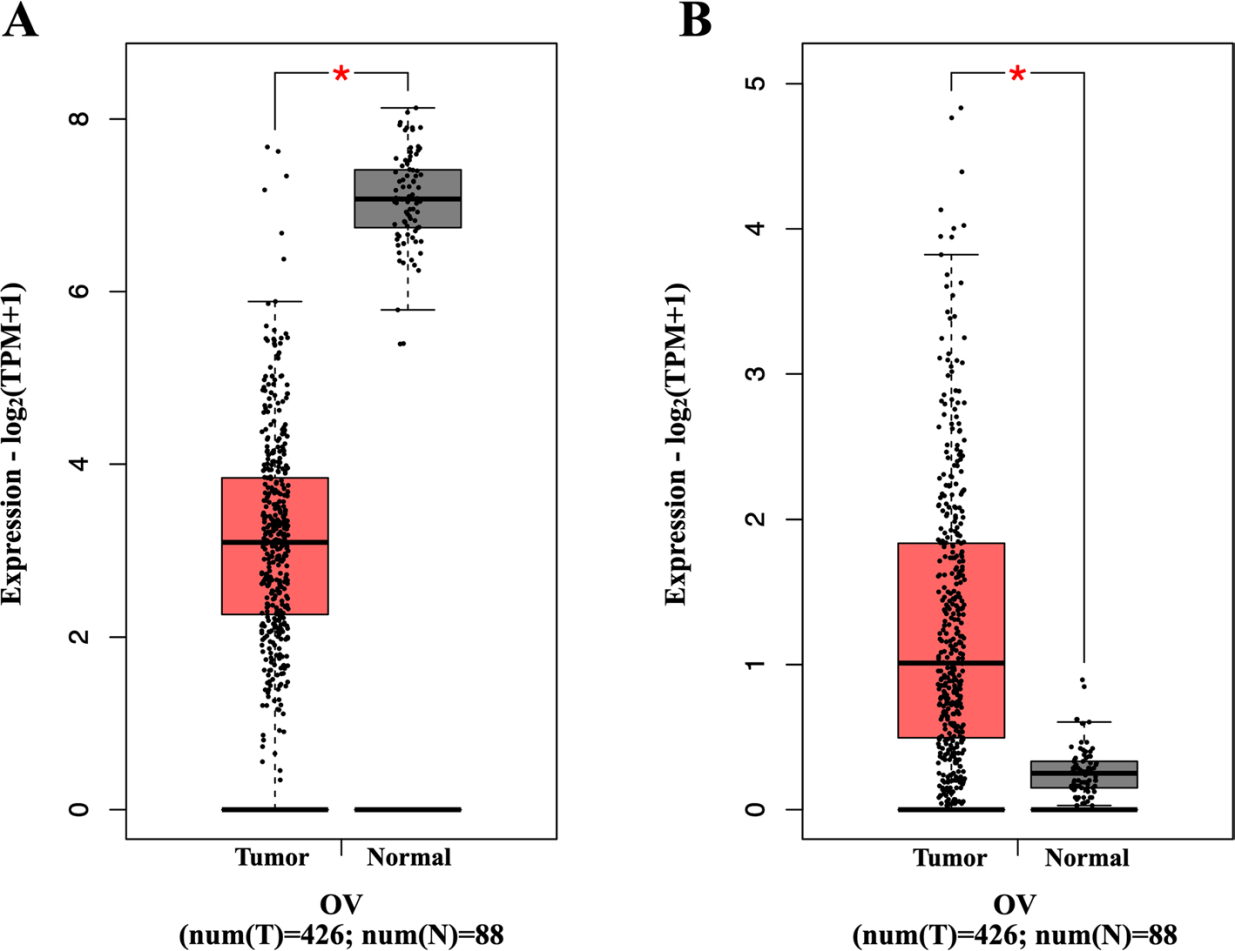
The mRNA levels of SALL4 and ALDH1A1 in serous ovarian carcinoma (SOC) on Gene Expression Profiling Interactive Analysis2 (GEPIA2). **A** Up-regulation of SALL4 and) down-regulation of ALDH1A1 expression in mRNA levels significantly for SOC compared with normal tissue by GEPIA2 were observed

**Fig. 2:**
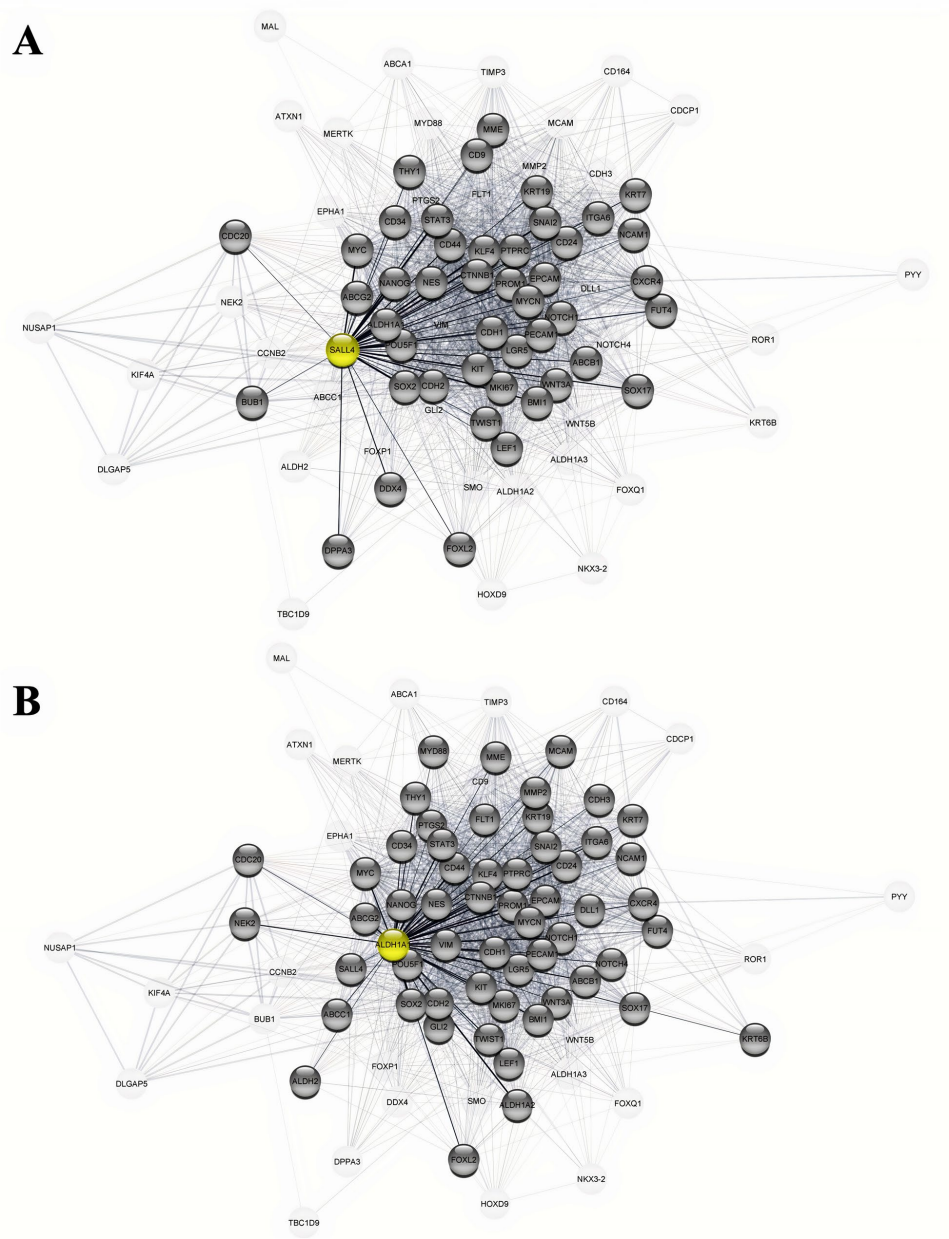
Protein-protein interaction (PPI) network for SALL4 and ALDH1A1 with other ovarian cancer stem cell (CSC) markers. **A** Interactions of SALL4 and **B** ALDH1A1 proteins with other ovarian CSC markers based on STRING databases via AtringApp using Cytoscape. An interaction with a low confidence (0.15) was observed between SALL4 and ALDH1A1 proteins

**Fig. 3:**
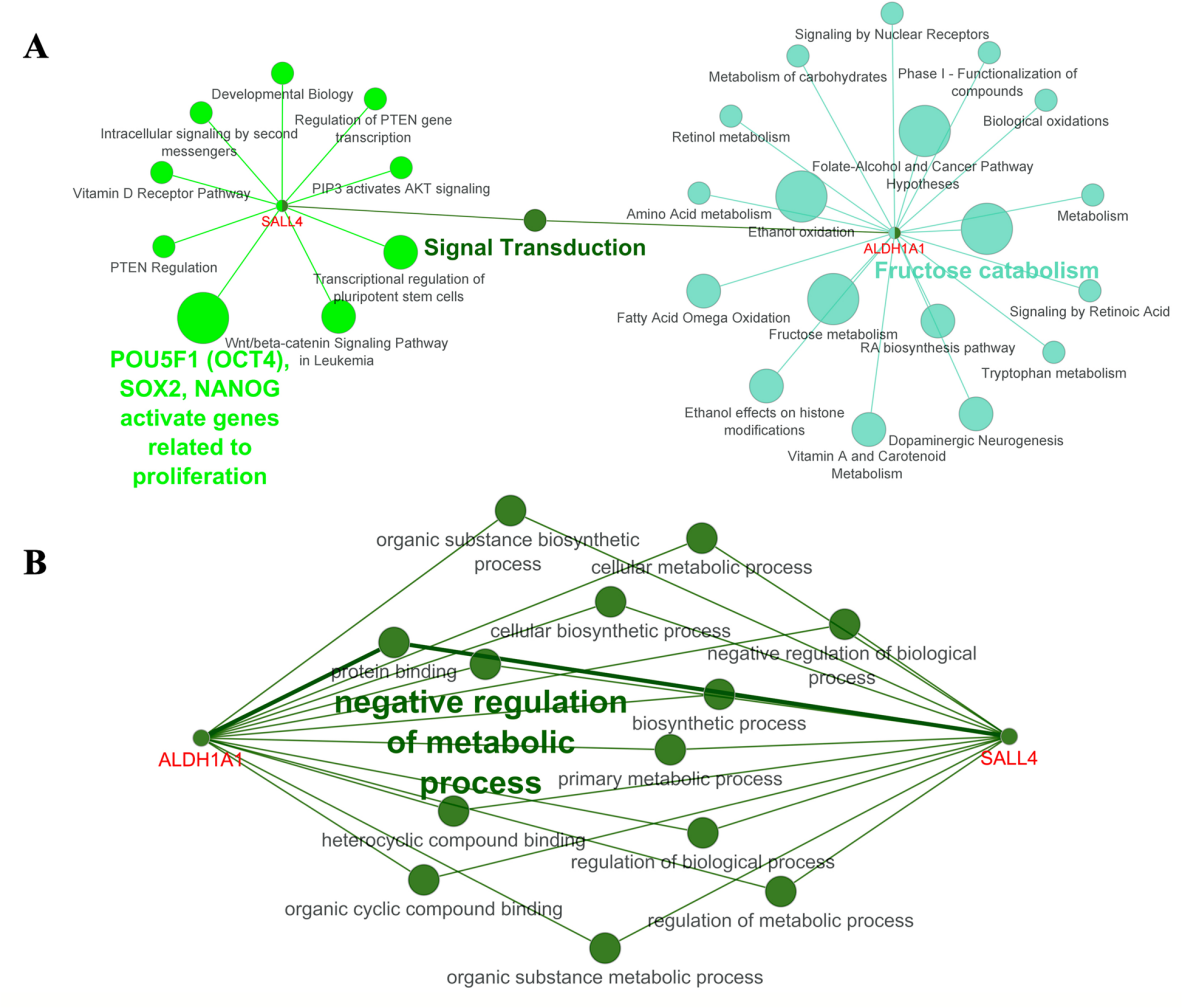
Pathway and gene ontology (GO) analysis for SALL4 and ALDH1A1using the ClueGO plugin in Cytoscape. **A** Pathway analysis, based on KEGG, Reactome, and Wikipathways as well as **B** common results of GO analysis for SALL4 and ALDH1A1 according to biological processes and molecular functional enrichment

To determine the expression level of SALL4 and ALDH1A1 in SOC, GEPIA2 was initially used. As shown in Fig. 1A, a significantly higher expression level of SALL4 was found in SOC tissues compared to the normal tissues. However, mRNA expression of ALDH1A1 indicated a significant reduction in tumor tissues rather than normal tissues by GEPIA2 tool analysis (Fig. 1B). Moreover, identification of connections between SALL4 and ALDH1A1 as well as with other CSC markers by PPI network were applied. These data indicated an interaction gene between SALL4 and ALDH1A1 (Fig.2). The PPI network showed that SALL4 and ALDHI1A1 have interactions with other important cancer stemness genes and CSC markers such as NANOG, POUF5 (OCT4), SOX2, CD44, and CD133 (PROM1) as shown in Fig.2 A and B. Additionally, genetic interaction between SALL4 and ALDH1A1 was observed by GeneMANIA (Supplementary Fig. 1). Enrichment analysis revealed pathways and common features of BP and MF about SALL4 and ALDH1A1 as demonstrated in Fig.3 A and B. According to these data, SALL4 was involved in PTEN regulation, and AKT signaling and activation genes were related to the proliferation pathway, while ALDH1A1 was associated with the metabolism pathways. In total, some of these pathways are based on the Reactome database as related to signal transduction.

### Characteristics of SOC patients

Forty-five paraffin-embedded SOC tissue samples have been included in this study. The mean age of the patients was 45 (SD = 14.8) years old, (ranged from 16 to 74) years old; 19 (42.2%) patients were younger than 45 years old, and 26 (57.8%) subjects were over 45 years old. The range of the tumor size was between 1 and 23 cm at the largest diameter, and tumors were classified into two groups based on the mean of tumor size (8 cm): Group 1: more than 8 cm or equal 8; 25 (55.6%) and Group 2: less than 8 cm; 11 (24.4%). Nine (20.0%) patients had no data on the tumor size. In the current study, 20 (44.4%) subjects had low histological grade (grade I), 4 (8.9%) had grade II, and 21 (46.7%) had high histological grade (grade III). Furthermore, 10 (22.2%) patients were at FIGO stage I, 15 (33.3%) were at stage II, and 20 (44.4%) were at FIGO stage III. LN and VI metastases were found in 20 (44.4%) and 5 (11.1%) subjects, respectively. Other cancer-involved regions were as follows: omentum with 9 (20.0%), fallopian tube with 9 (20.0%), cervix with 8 (17.8%), endometrium with 5 (11.1%), myometrium with 6 (13.3%), vagina with 10 (22.2%), peritoneum with 3 (6.7%), lymphovascular space invasion with 8 (17.8%), perineural invasion with 3 (6.7%), colon with 10 (22.2%), small intestine with 4 (8.9%), post-cul-de-sac with 7 (15.6%) and paracolic lymph node with 5 (11.1%). Moreover, tumor recurrence and distant metastasis were detected in 25 (55.6%) and 20 (44.4%) patients, respectively.

### Expression of SALL4 and ALDH1A1 in SOC, benign tumors, and normal specimens

**Fig. 4:**
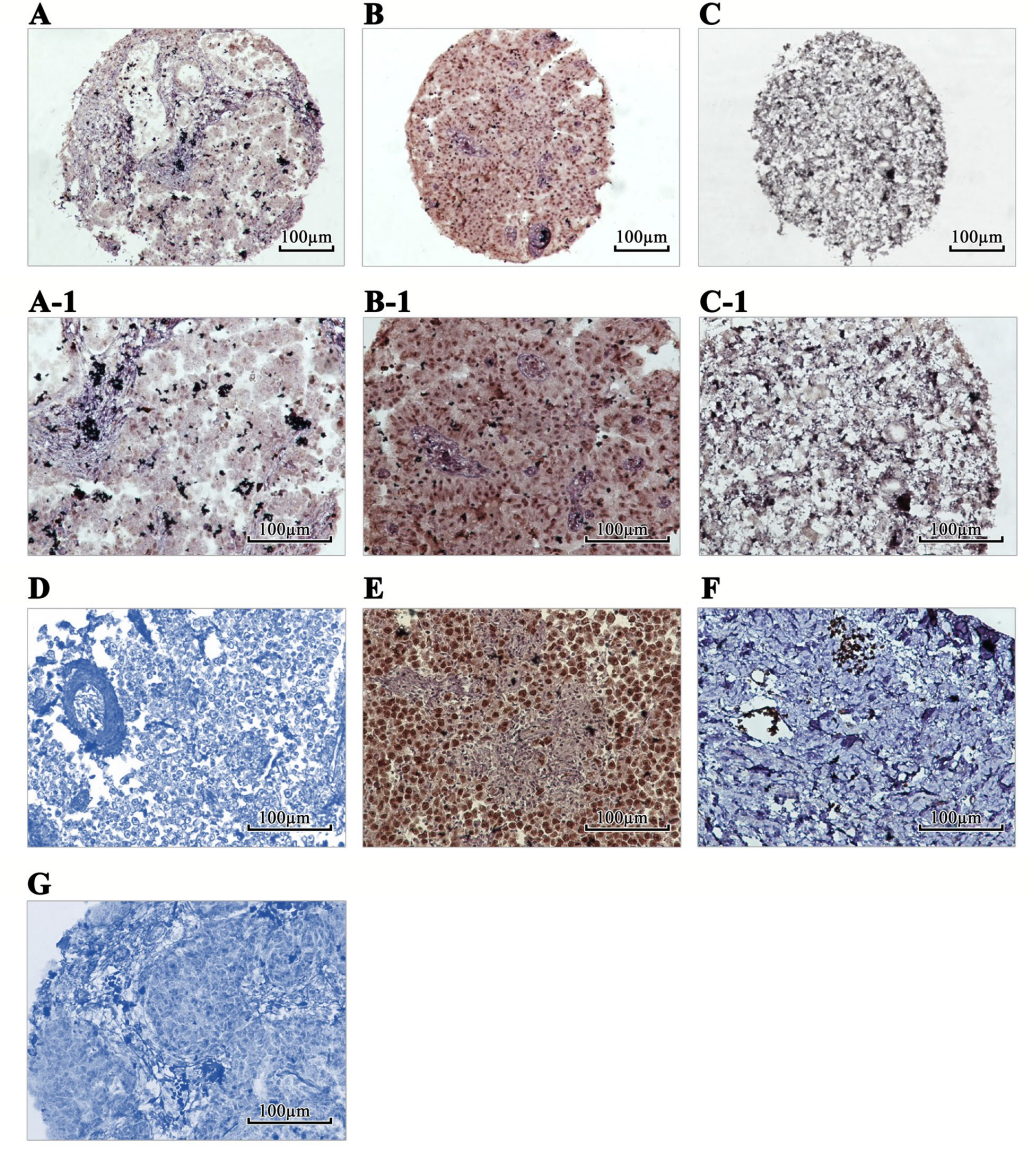
Immunohistochemical staining of SALL4 protein in serous ovarian carcinoma (SOC) patients, benign tumors, and normal tissues. (A, A-1): Low nuclear expression of SALL4 in patients with SOC. (B, B-1): High nuclear SALL4 expression in SOC patients. (C, C-1): Expression of SALL4 was found in nucleus in benign tumor. Human germ cell tumor of testis tissues (Seminoma) as controls (D): negative and (E): positive. (F): Expression of SALL4 was not observed in normal ovarian tissue samples. (G): Isotype control. Figures have magnification of 100 × and 200 ×

**Fig. 5:**
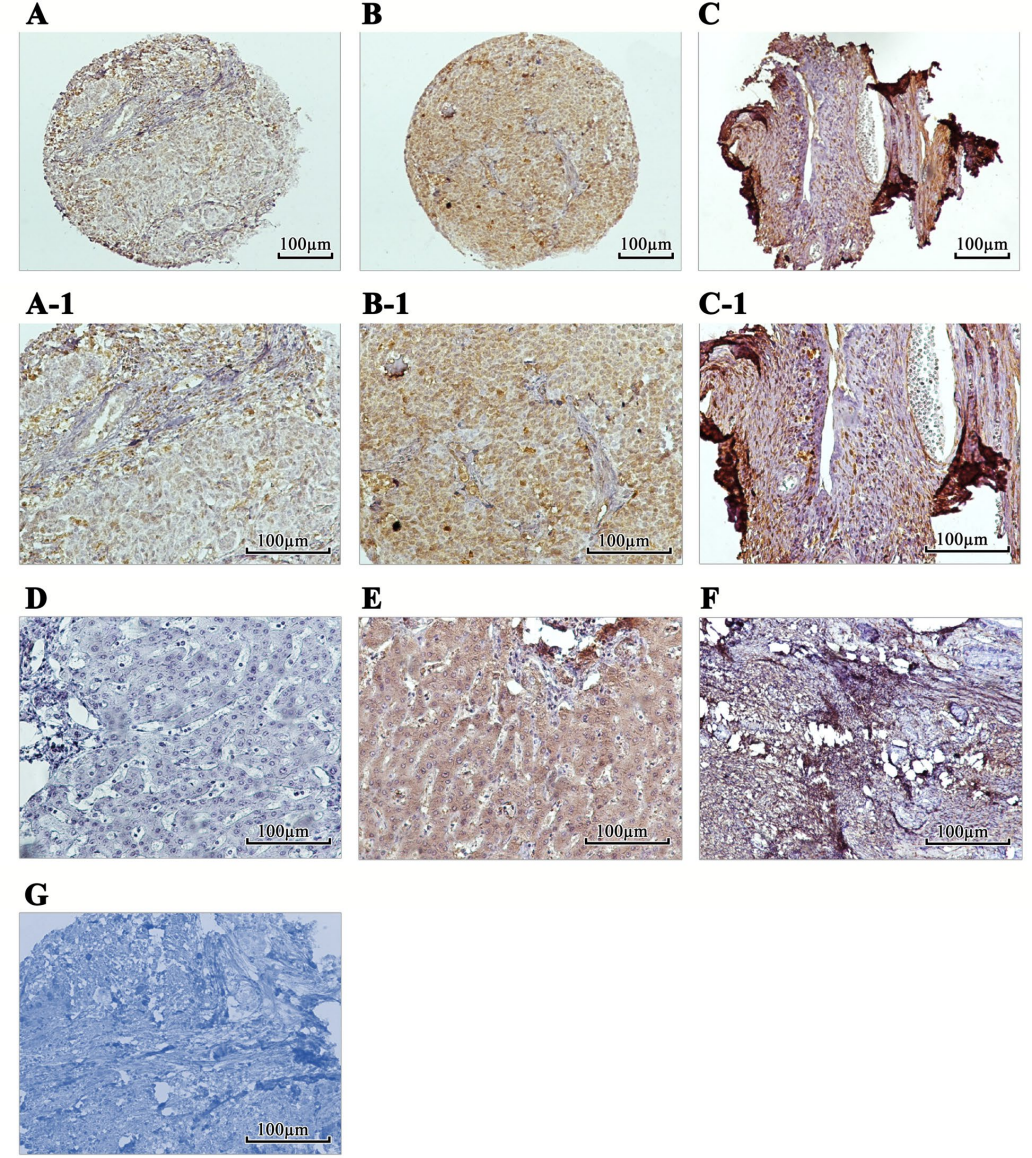
Immunohistochemical staining of ALDH1A1 protein in serous ovarian carcinoma (SOC) patients, benign tumors, and normal tissues. (A-A-1): Low cytoplasmic expression of ALDH1A1 in patients with SOC. (B-B-1): High cytoplasmic expression of ALDH1A1 was observed in SOC patients. (C-C-1): High ALDH1A1 expression was observed in benign tumors rather than tumor tissues. Human normal liver tissues as controls (D): negative and (E): positive. (F): Expression of ALDH1A1 was higher in normal ovarian tissue samples in comparison to benign tumors. (G): Isotype control. Figures have magnification of 100 × and 200×

To evaluate the expression pattern and clinical significance of SALL4 and ALDH1A1, their expression level was analyzed in a set of 45 paraffin-embedded SOC tissue samples using the IHC technique on TMA slides and by applying three scoring methods, including intensity of staining, percentage of positive tumor cells, and H-score (Table 1). The expression level of SALL4 was evaluated in benign tumors and normal tissue samples. Positive staining of SALL4 was mainly observed in the nucleus of the SOC tissue. Importantly, the expression level of SALL4 was significantly higher in SOC tissues compared to benign and normal samples (Fig.4). Also, positive staining of ALDH1A1 was detected in the cytoplasm of SOC. The expression level of ALDH1A1 was significantly lower in SOC tissues compared to benign tumors and normal tissue samples (Fig. 5).

**Table 1:** Expression of SALL4 and ALDH1A1 (Intensity of staining, percentage of positive tumor cells, and H-score) in serous ovarian carcinoma (SOC), benign ovarian tumors, and normal tissue samples

Scoring system	Serous ovarian carcinoma N (%) SALL4	Benign ovarian tumors N (%) SALL4	Normal Samples N (%) SALL4	*P-value*	Serous ovarian carcinoma N (%) ALDH1A1	Benign ovarian tumors N (%) ALDH1A1	Normal Samples N (%) ALDH1A1	*P-value*
Intensity of staining								
Negative (0)	12 (26.7)	35 (94.6)	20 (100.0)	***< 0.001***	14 (31.1)	4 (26.7)	0 (0.0)	***0.048***
Weak (+ 1)	20 (44.4)	1 (2.7)	0 (0.0)		8 (17.8)	1 (6.7)	3 (15.0)	
Moderate (+ 2)	9 (20.0)	1 (2.7)	0 (0.0)		4 (8.9)	2 (13.3)	7 (35.0)	
Strong (+ 3)	4 (8.9)	0 (0.0)	0 (0.0)		19 (42.2)	8 (53.3)	10 (50.0)	
Percentage of positive tumor cells								
< 25%	24 (53.3)	35 (94.6)	0 (0.0)	***< 0.001***	21 (46.7)	5 (33.3)	0 (0.0)	0.254
25–50%	10 (22.2)	1 (2.7)	0 (0.0)		4 (8.9)	0 (0.0)	2 (10.0)	
51- 75%	3 (6.7)	1 (2.7)	0 (0.0)		1 (2.2)	0 (0.0)	1 (5.0)	
> 75%	8 (17.8)	0 (0.0)	20 (100.0)		19 (42.2)	10 (66.7)	17 (85.0)	
H-score cut off =100								
Low	33 (73.3)	35 (94.6)	0 (0.0)	***< 0.001***	25 (55.6)	5 (33.3)	10 (50.0)	***0.050***
High	12 (26.7)	2 (5.4)	0 (0.0)		20 (44.4)	10 (66.7)	10 (50.0)	
Total	45	37	20		45	15	20	

*H-score* histological score

*P value* is based on Kruskal-Wallis & Mann-Whitney *U* tests

Values in bold are statistically significant

### Associations between the co-expression of SALL4/ALDH1A1 and clinicopathological features

In the current study, the association between the co-expression of SALL4/ALDH1A1 proteins with clinicopathological parameters was examined through Pearson’s chi-square test. The expression levels of SALL4 /ALDH1A1 were divided into two categories based on mean expression and four phenotypes including SALL4 ^High^/ ALDH1A1^High^, SALL4 ^High^/ ALDH1A1^Low^, SALL4 ^Low^/ ALDH1A1^High^, and SALL4 ^Low^/ ALDH1A1 ^Low^ (Table 2). The results of analysis showed that a highly significant association between co-expression of SALL4/ALDH1A1 proteins and advanced FIGO stage (*P = 0.047)*. The results also revealed that SALL4 ^High^/ ALDH1A1^Low^ phenotype was found more in patients with FIGO stage III. Moreover, there was a statistically significance association between the co-expression of SALL4/ALDH1A1 proteins and distant metastasis (*P = 0.028)* (Table 2).

**Table 2:** The association between co-expression of SALL4/ALDH1A1 and clinicopathological parameters of serous ovarian carcinoma (SOC) samples (Intensity of staining and H-score) (*P value*; Pearson’s χ2 test)

Patients and tumor characteristics	Total samples N (%)	SALL4/ALDH1 phenotypes				*P-value*
		SALL4 ^Low^ /ALDH1A1 ^Low^	SALL4 ^Low^ /ALDH1A1 ^High^	SALL4 ^High^ /ALDH1A1 ^Low^	SALL4 ^High^ /ALDH1A1 ^High^	
OSC	45 (100.0)	19 (42.2)	14 (31.1)	6 (13.3)	6 (13.3)	
Mean age, years (Range)	45 (16-74)					
≤Mean age	19 (42.2)	7 (36.8)	5 (35.7)	2 (33.3)	5 (83.3)	0.185
>Mean age	26 (57.8)	12 (63.2)	9 (64.3)	4 (66.7)	1 (16.7)	
Mean tumor size (cm)	8.28					
≤Mean	25 (55.6)	11 (78.6)	9 (75.0)	3 (75.0)	2 (33.3)	0.215
>Mean	11 (24.4)	3 (21.4)	3 (25.0)	1 (25.0)	4 (66.7)	
Not identified	9 (20.0)	0 (0.0)	0 (0.0)	0 (0.0)	0 (0.0)	
Histological grade						
I	20 (44.4)	9 (47.4)	7 (50.0)	0 (0.0)	4 (66.7)	0.359
II	4 (8.9)	2 (10.5)	1 (7.1)	1 (16.7)	0 (0.0)	
III	21 (46.7)	8 (42.1)	6 (42.9)	5 (83.3)	2 (33.3)	
IV	0 (0.0)	0 (0.0)	0 (0.0)	0 (0.0)	0 (0.0)	
FIGO stage						
I	10 (22.2)	5 (26.3)	3 (21.4)	0 (0.0)	2 (33.3)	***0.047***
II	15 (33.3)	5 (26.3)	7 (50.0)	0 (0.0)	3 (50.0)	
III	20 (44.4)	9 (47.4)	4 (28.6)	6 (100.0)	1 (16.7)	
IV	0 (0.0)	0 (0.0)	0 (0.0)	0 (0.0)	0 (0.0)	
Lymph node (LN) metastasis						
Involved	20 (44.4)	9 (50.0)	6 (50.0)	2 (33.3)	3 (50.0)	0.903
None	22 (48.9)	9 (50.0)	6 (50.0)	4 (66.7)	3 (50.0)	
Not identified	3 (6.7)	0 (0.0)	0 (0.0)	0 (0.0)	0 (0.0)	
Vascular invasion (VI)						
Involved	5 (11.1)	1 (5.6)	1 (8.3)	2 (33.3)	1 (16.7)	0.309
None	37 (82.2)	17 (94.4)	11(91.7)	4 (66.7)	5 (83.3)	
Not identified	3 (6.7)	0 (0.0)	0 (0.0)	0 (0.0)	0 (0.0)	
Omentum						
Involved	9 (20.0)	5 (27.8)	1 (8.3)	2 (33.3)	1 (16.7)	0.524
None	33 (73.3)	13 (72.2)	11 (91.7)	4 (66.7)	5 (83.3)	
Not identified	3 (6.7)	0 (0.0)	0 (0.0)	0 (0.0)	0 (0.0)	
Fallopian tube						
Involved	9 (20.0)	4 (22.2)	3 (25.0)	2 (33.3)	0 (0.0)	0.524
None	33 (73.3)	14 (77.8)	9 (75.0)	4 (66.7)	6 (100.0)	
Not identified	3 (6.7)	0 (0.0)	0 (0.0)	0 (0.0)	0 (0.0)	
Cervix						
Involved	8 (17.8)	5 (27.8)	1 (8.3)	2 (33.3)	0 (0.0)	0.263
None	34 (75.6)	13 (72.2)	11 (91.7)	4 (66.7)	6 (100.0)	
Not identified	3 (6.7)	0 (0.0)	0 (0.0)	0 (0.0)	0 (0.0)	
Endometrium						
Involved	5 (11.1)	3 (16.7)	1 (8.3)	1 (16.7)	0 (0.0)	0.688
None	37 (82.2)	15 (83.3)	11 (91.7)	5 (83.3)	6 (100.0)	
Not identified	3 (6.7)	0 (0.0)	0 (0.0)	0 (0.0)	0 (0.0)	
Myometrium						
Involved	6 (13.3)	3 (16.7)	1 (8.3)	1 (16.7)	1 (16.7)	0.922
None	36 (80.0)	15 (83.3)	11 (91.7)	5 (83.3)	5 (83.3)	
Not identified	3 (6.7)	0 (0.0)	0 (0.0)	0 (0.0)	0 (0.0)	
Vagina						
Involved	10 (22.2)	3 (16.7)	3 (25.0)	3 (50.0)	1 (16.7)	0.399
None	32 (71.1)	15 (83.3)	9 (75.0)	3 (50.0)	5 (83.3)	
Not identified	3 (6.7)	0 (0.0)	0 (0.0)	0 (0.0)	0 (0.0)	
Peritoneum						
Involved	3 (6.7)	2 (10.5)	1 (8.3)	0 (0.0)	0 (0.0)	0.728
None	40 (88.9)	17 (89.5)	11 (91.7)	6 (100.0)	6 (100.0)	
Not identified	2 (4.4)	0 (0.0)	0 (0.0)	0 (0.0)	0 (0.0)	
Lymphovascular space invasion						
Involved	8 (17.8)	4 (21.1)	3 (25.0)	1 (16.7)	0 (0.0)	0.618
None	35 (77.8)	15 (78.9)	9 (75.0)	5 (83.3)	6 (100.0)	
Not identified	2 (4.4)	0 (0.0)	0 (0.0)	0 (0.0)	0 (0.0)	
Perineural invasion						
Present	3 (6.7)	0 (0.0)	2 (16.7)	1 (16.7)	0 (0.0)	0.230
Absent	39 (86.7)	18 (100.0)	10 (83.3)	5 (83.3)	6 (100.0)	
Not identified	3 (6.7)	0 (0.0)	0 (0.0)	0 (0.0)	0 (0.0)	
Colon						
Involved	10 (22.2)	4 (22.2)	3 (23.1)	3 (50.0)	0 (0.0)	0.237
None	33 (73.3)	14 (77.8)	10 (76.9)	3 (50.0)	6 (100.0)	
Not identified	2 (4.4)	0 (0.0)	0 (0.0)	0 (0.0)	0 (0.0)	
Small intestine						
Involved	4 (8.9)	3 (16.7)	1 (8.3)	0 (0.0)	0 (0.0)	0.503
None	38 (84.4)	15 (83.3)	11 (91.7)	6 (100.0)	6 (100.0)	
Not identified	3 (6.7)	0 (0.0)	0 (0.0)	0 (0.0)	0 (0.0)	
Post-cul-de-sac						
Involved	7 (15.6)	5 (27.8)	0 (0.0)	2 (33.3)	0 (0.0)	0.094
None	35 (77.8)	13 (72.2)	12 (100.0)	4 (66.7)	6 (100.0)	
Not identified	3 (6.7)	0 (0.0)	0 (0.0)	0 (0.0)	0 (0.0)	
Paracolic lymph node						
Involved	5 (11.1)	4 (22.2)	1 (8.3)	0 (0.0)	0 (0.0)	0.309
None	37 (82.2)	14 (77.8)	11 (91.7)	6 (100.0)	6 (100.0)	
Not identified	3 (6.7)	0 (0.0)	0 (0.0)	0 (0.0)	0 (0.0)	
Distant metastasis						
Present	20 (44.4)	6 (31.6)	6 (42.9)	6 (100.0)	2 (33.3)	***0.028***
Absent	25 (55.6)	13 (68.4)	8 (57.1)	0 (0.0)	4 (66.7)	
Tumor recurrence						
Yes	25 (55.6)	10 (52.6)	6 (42.9)	6 (100.0)	3 (50.0)	0.119
No	20 (44.4)	9 (47.4)	8 (57.1)	0 (0.0)	3 (50.0)	

### Clinical outcomes in patients with SOC

The mean and median follow-up times for DSS were (32; SD = 18.1 and 24; Q1, Q3 = 22, 45) months or PFS (26; SD = 19.9 and 24; Q1, Q3 = 12, 30), respectively. The minimum, maximum, and range of these follow-up times for DSS were 5, 84, and 79 months, and for PFS, they were 1, 84, and 83 months, respectively. During the follow-up period, cancer-related deaths were found in 16 patients (35.6%). Our results indicated that tumor recurrence and metastasis have occurred in 25 (55.6%) and 20 (44.4%) cases, while 20 (44.4%) and 25 (55.6%) subjects showed negative results for the above-mentioned parameters, respectively. Additionally, 26 (57.8%) patients were positive for both tumor recurrence and metastasis, but 19 (42.2%) patients were negative for these two features.

### Survival outcomes of DSS or PFS based on the co-expression of SALL4/ALDH1A1

**Fig. 6:**
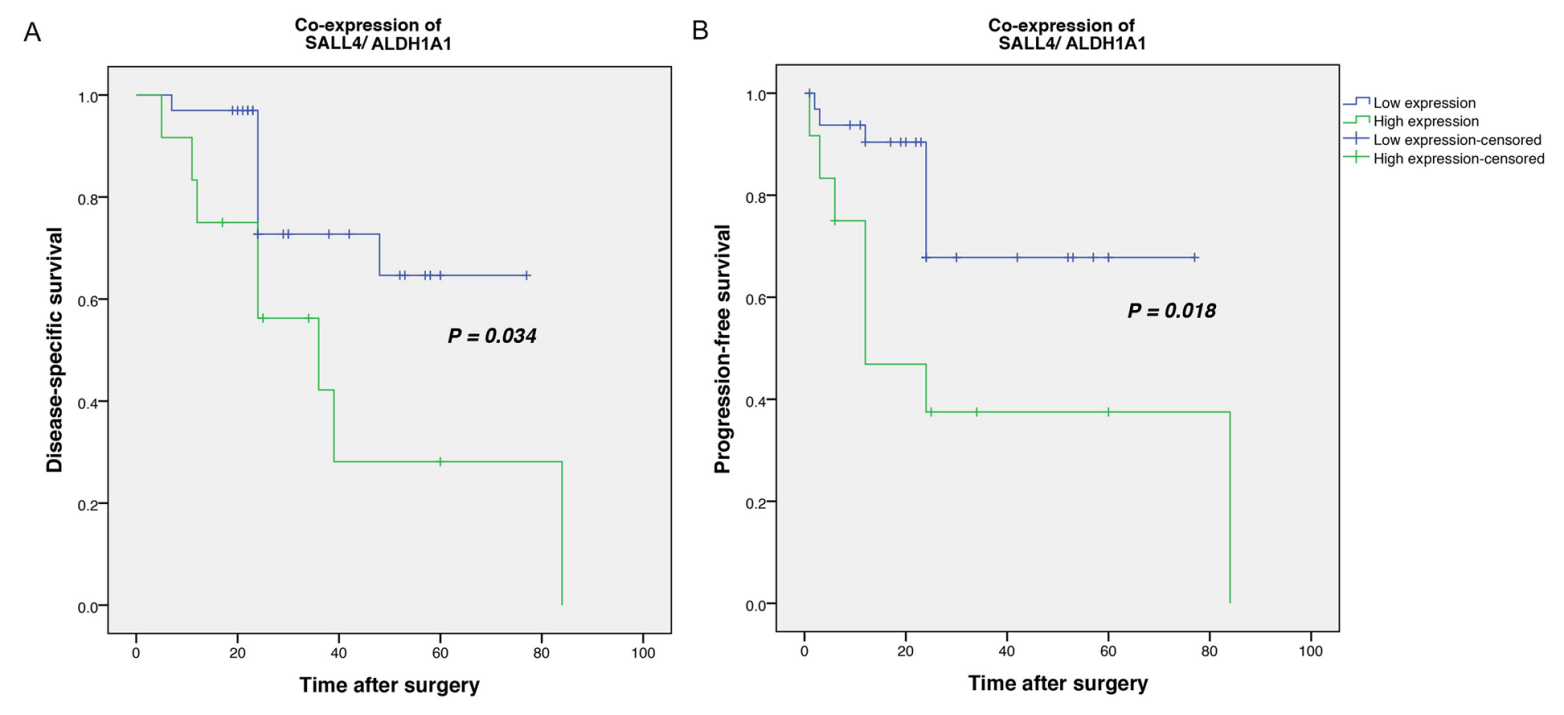
Kaplan–Meier survival curves for disease-specific survival (DSS) and progression-free survival (PFS) according to the co-expression levels of SALL4/ALDH1A1 proteins in serous ovarian carcinoma (SOC) patients. A high level of the co-expression of SALL4/ALDH1A1 proteins was found to be significantly associated with shorter DSS (*P = 0.034)* (A) and PFS (*P = 0.018)* (B) in patients with SOC

Kaplan–Meier survival analysis was used to compare DSS or PFS based on the co-expression of SALL4/ALDH1A1 (H-score) in SOC samples. In this study, SALL4 ^High^/ ALDH1A1^High^ and SALL4 ^High^/ ALDH1A1^Low^ were classified as a high co-expression group and SALL4 ^Low^/ ALDH1A1^High^ and SALL4 ^Low^/ ALDH1A1 ^Low^ as a low co-expression group. The mean DSS and PFS time for the patients whose specimens expressed high and low co-expression of SALL4/ALDH1A1 were obtained as 41 (SD = 10.0) and 59 (SD = 5.0), and 37 (SD = 11.6) and 58 (SD = 5.5) months, respectively. The Kaplan–Meier survival analysis indicated significant differences between DSS (Log-rank test; *P = 0.034*) or PFS (Log-rank test; *P = 0.018*) and the patients with high and low co-expression of SALL4/ALDH1A1 (Fig.6 A, B). Moreover, the 5-year survival rate for DSS or PFS of the patients who showed high SALL4/ALDH1A1 was 58 and 35% and in those with low was 27 and 62% (*P = 0.047*, *P = 0.012*), respectively.

Univariate and multivariate analyses were also applied to assess the clinical significance of various parameters that might affect DSS or PFS in patients with SOC. As shown in Tables 3 and 4, the histological grades (*P = 0.045*), particularly grade III versus grade I, was significant risk factor: affecting the DSS and PFS of SOC patients in the univariate analysis. Furthermore, histological grades added prognostic value in grade III versus grade I of patients with SOC in PFS in multivariate analysis. Some other variables, including VI metastasis, vagina involvement, cervix involvement, myometrium involvement, post-cul-de-sac involvement, metastasis, and tumor recurrence had *p*-values of less than 0.05; however, hazard ratio (HR) was not more than 1 (Tables 3 and 4). Therefore, these variables were not included in the multivariate analysis. Moreover, the co-expression of SALL4/ALDH1A1 (HR: 4.095, 95% CI: (1.295- 12.952); *P = 0.016*) was found as an independent prognostic factor affecting the PFS in the multivariate analysis in OSC patients (Table 4). Notably, the other clinicopathological variables were not significant factors for DSS or PFS in multivariate analyses.

**Table 3:** Univariate and multivariate Cox regression analyses of potential prognostic factor for disease-specific survival (DSS) in patients with serous ovarian carcinoma

Covariate	Univariate analysis		Multivariate analysis	
	HR (95% CI)	***P-value***	HR (95% CI)	***P-value***
SALL4/ALDH1A1 coexpression High versus Low	2.689 (0.974- 7.426)	0.056	2.578 (0.909- 7.311)	0.075
Histological grade		***0.045***		0.071
II versus I	0.205 (0.120- 2.228)	0.970	0.100 (0.020- 2.20)	0.968
III versus I	5.002 (1.409- 17.751)	***0.013***	4.471 (1.249- 16.010)	***0.021***
VI metastasis	0.265 (0.079- 0.886)	***0.031***	–	–
Vagina involvement	0.319 (0.103- 0.993)	***0.049***	–	–
Cervix involvement	0.214 (0.067- 0.687)	***0.010***	–	–
Myometrium involvement	0.290 (0.092- 0.916)	***0.035***	–	–
Post-cul-de-sac involvement	0.262 (0.083- 0.830)	***0.023***	–	–
Distant metastasis	0.114 (0.026- 0.507)	***0.004***	–	–
Tumor recurrence	0.015 (0.000- 0.985)	***0.049***	–	–

*H-score* histological score

Values in bold are statistically significant

The variables with *P* value less than 0.05 and HR more than 1.0 were included in multivariable analyses *HR* hazard ratio, *CI* confidence interval

**Table 4:** Univariate and multivariate Cox regression analyses of potential prognostic factor for progression-free survival (PFS) in patients with serous ovarian carcinoma

Covariate	Univariate analysis		Multivariate analysis	
	HR (95% CI)	***P-value***	HR (95% CI)	***P-value***
SALL4/ ALDH1A1				
coexpression High versus Low	3.018 (1.091- 8.344)	***0.033***	4.095 (1.295- 12.952)	***0.016***
Histological grade		***0.025***		0.770
II versus I	1.863 (0.192- 18.045)	0.974	0.010 (0.100- 2.222)	0.964
III versus I	5.848 (1.636- 20.899)	***0.007***	1.922 (0.326- 11.348)	0.471
FIGO stage		***0.017***		0.192
II versus I	0.575 (0.081- 4.090)	0.581	0.601 (0.077- 4.677)	0.627
III versus I	3.921 (0.867- 17.737)	0.076	3.501 (608- 20.175)	0.161
Vascular invasion (VI)	0.286 (0.086- 0.956)	***0.042***	–	–
Cervix involvement	0.182 (0.057- 0.582)	***0.004***	–	–
Myometrium involvement	0.207 (0.065- 0.656)	***0.007***	–	–
Vagina involvement	0.250 (0.080- 0.777)	***0.017***	–	–
Colon involvement	0.314 (0.105- 0.934)	***0.037***	–	–
Post-cul-de-sac involvement	0.177 (0.054- 0.579)	***0.004***	–	–
Distant metastasis	0.111 (0.025- 0.490)	***0.004***	–	–
Tumor recurrence	0.012 (0.000- 0.810)	***0.040***	–	**–**

*H-score* histological score

Values in bold are statistically significant

The variables with *P* value less than 0.05 and HR more than 1.0 were included in multivariable analyses *HR* hazard ratio, *CI* confidence interval

## Discussion

Despite advances in screening for early diagnosis and treatment of ovarian cancer patients, it is still one of the deadliest cancers among gynecologic tumors [53]. Therefore, finding new biomarkers for prognostic or selecting appropriate treatment is crucial.

In our data from in-silico analysis, significant differential mRNA expression of SALL4 and ALDH1A1 were observed in SOC tissues in comparison to normal tissues using bioinformatics analysis. These data indicated up-regulation of SALL4 and down-regulation of ALDH1A1 expression in mRNA levels. Expectedly based on these data, our experiment on SOC tissue samples demonstrated higher expression of SALL4 in tumor tissues rather than normal tissue samples while the expression of ALDH1A1 protein reduced in tumor tissues in comparison to normal tissues. Previous clinical research in ovarian cancer patients indicated overexpression of SALL4 protein [14, 27] while conflicting expression patterns for ALDH1A1 at the protein level have been reported in ovarian cancer studies [37, 38]. Nevertheless, our finding about ALDH1A1 protein was consistent with previous evidence that indicated reduced ALDH1A1 staining compared to normal in SOC [54].

Earlier investigations on ovarian cancer have focused on the analysis and characterization of the expression of these two markers, separately [26, 37] while there are no studies of the combinations of SALL4 and ALDH1 in ovarian cancer. However, there is association between SALL4 and ALDH1 in other types of cancer by in-vitro and in-silico evidence [15, 16]. In this regard, Kong et al. found that knock-down SALL4 gene in liver cancer cells led to lower expression of ALDH1A1 [16].

In this research, PPI network and GeneMANIA analysis demonstrated the weakness relationships as genetic interaction between SALL4 and ALDH1A1. Also, common interactions of these two markers with CSC markers such as stemness and EMT markers encouraged the investigation of the co-expression of SALL4 and ALDH1A1 proteins. To the best of our knowledge, the prognostic significance of SALL4/ALDH1A1 in SOC patients remains largely unknown. Therefore, in the present study, the clinical significance and prognostic value of the co-expression of SALL4/ALDH1A1 were investigated with various clinicopathological features and survival outcomes by applying the IHC technique on TMA sections in patients with SOC, benign tumors, and normal ovarian samples.

IHC analysis of SOC tissues compared to benign tumors and normal tissue samples indicated that SALL4 protein expression is upregulated in SOCs in comparison to benign tumors and normal tissues. However, lower expression of ALDH1A1 was evident in SOC samples rather than benign tumors and normal tissues. Previous studies have shown that SALL4 and ALDH1A1 are expressed in primordial germ cells (PGCs) and very small embryonic-like stem cell (VSEL) in normal ovary tissue samples [55]. On the other hand, higher expression of ALDH1 in VSELs have been reported in ovarian cancer tissues [56].

We found, for the first time, a statistically significant association between the increased co-expression of SALL4/ALDH1A1 and the advanced FIGO stage as well as distant metastasis. However, in preliminary studies, positive association of SALL4 and ALDH1A1 expression proteins were reported separately with advanced FIGO stage in ovarian cancer tissue samples [14, 57].

Cancer studies have investigated roles and functions of these two CSC markers separately in tumor progression, invasiveness and metastases so that high expression of SALL4 and low expression of ALDH1A1 are associated with advanced levels of the disease [14, 58].

In our data, histological grade and FIGO stage were observed as prognostic factors for PFS in univariate analysis. Moreover, our findings demonstrated that histological grade can be considered as a prognostic factor in univariate analysis, and that the co-expression of SALL4/ALDH1A1 added prognostic values of DSS in patients with SOC who had grade III versus grade I in multivariate analysis. It can be concluded that the increased co-expression of SALL4/ALDH1A1 is associated with tumor aggressiveness in these cases. Most notably, our results are in parallel with previous investigations that the high histological grade and the advanced tumor stage lead to tumor progression, metastasis and poorer clinical outcomes in cancer patients [59, 60]. Our findings have also shown that patients with high co-expression of SALL4/ALDH1A1 have a worse prognosis for DSS or PFS. The SOC patients with higher co-expression of SALL4/ALDH1A1 in their tumors indicated a shorter 5-year survival rate for DSS or PFS. Moreover, elevated co-expression of SALL4/ALDH1A1 proteins was recognized as a significant risk factor affecting the PFS in the univariate and multivariate analysis, and the co-expression of SALL4/ALDH1A1 was found as an independent prognostic factor of PFS.

To our recent knowledge, cancer studies have investigated the co-expression of CSC markers such as SALL4 or ALDH family with other CSC. The co-expression of ALDH/CD133 was recognized as an independent prognostic factor for the survival in ovarian cancer patients [12, 61]. Moreover, the co-expression of SALL4 and EpCAM was found to be significantly associated with poorer OS in HCC [62]. According to these findings, some co-expression of double or more CSC markers can display a more aggressive phenotype in cancer cells [63]. As a result, investigation of the co-expression of these CSC markers may predict tumor progression and advanced disease. The findings of our study were in line with those of earlier studies. Moreover, in this study, the co-expression of these two CSC markers led to deeper understanding of the prognostic values of SALL4/ALDH1A1 in the SOC patients. Interestingly, our findings emphasized the hypothesis that SALL4 combined with ALDH1A1 was more effective for the prognosis than the single marker in patients with SOC.

A limitation in our study was the description of the mechanism of this relationship in ovarian cancer cells, so a larger sample can lead to more generalizable results.

## Conclusions

In conclusion, a direct significant association was found between increased co-expression of SALL4/ALDH1A1 proteins with advanced FIGO stage and distant metastasis in the SOC patients. Moreover, we found that the co-expression of SALL4/ALDH1A1 proteins is associated with more aggressive tumor behavior, more advanced disease, and poor DSS, or PFS in SOC cases. Furthermore, higher co-expression of SALL4/ALDH1A1 proteins was found as an independent prognostic factor for PFS. Our finding confirmed that the combination of SALL4 with ALDH1A1 was a more effective biomarker for prognosis than the individual marker, particularly in ALDH1A1 in these cases. Furthermore, the co-expression of SALL4/ALDH1A1 may be a valuable biomarker in predicting the clinical outcome of patients with SOC. Further investigations with more patients are needed to verify our results.

## Supplementary Information

**Additional file 1: Supplementary Figure 1**. Network analysis based on GeneMANIA prediction server for SALL4 and ALDH1A1. GeneMANIA analysis indicated gene sets that were enriched in the target network of SALL4 and ALDH1A1. Physical Interactions and Genetic are shown by distinct colors of the network edge for gene sets.

**Additional file 2.** (XLS 29 kb)

## Data Availability

All data generated or analyzed during this study are included in this article and the raw data are available from the corresponding author on reasonable request.
