# Supplementary figures and images for: Co-expression of cancer stem cell markers, SALL4/ALDH1A1, is associated with tumor aggressiveness and poor survival in patients with serous ovarian carcinoma
Source: J Ovarian Res. 2022 Jan 28;15:17. doi: 10.1186/s13048-021-00921-x (PMC8800292; doi:10.1186/s13048-021-00921-x)

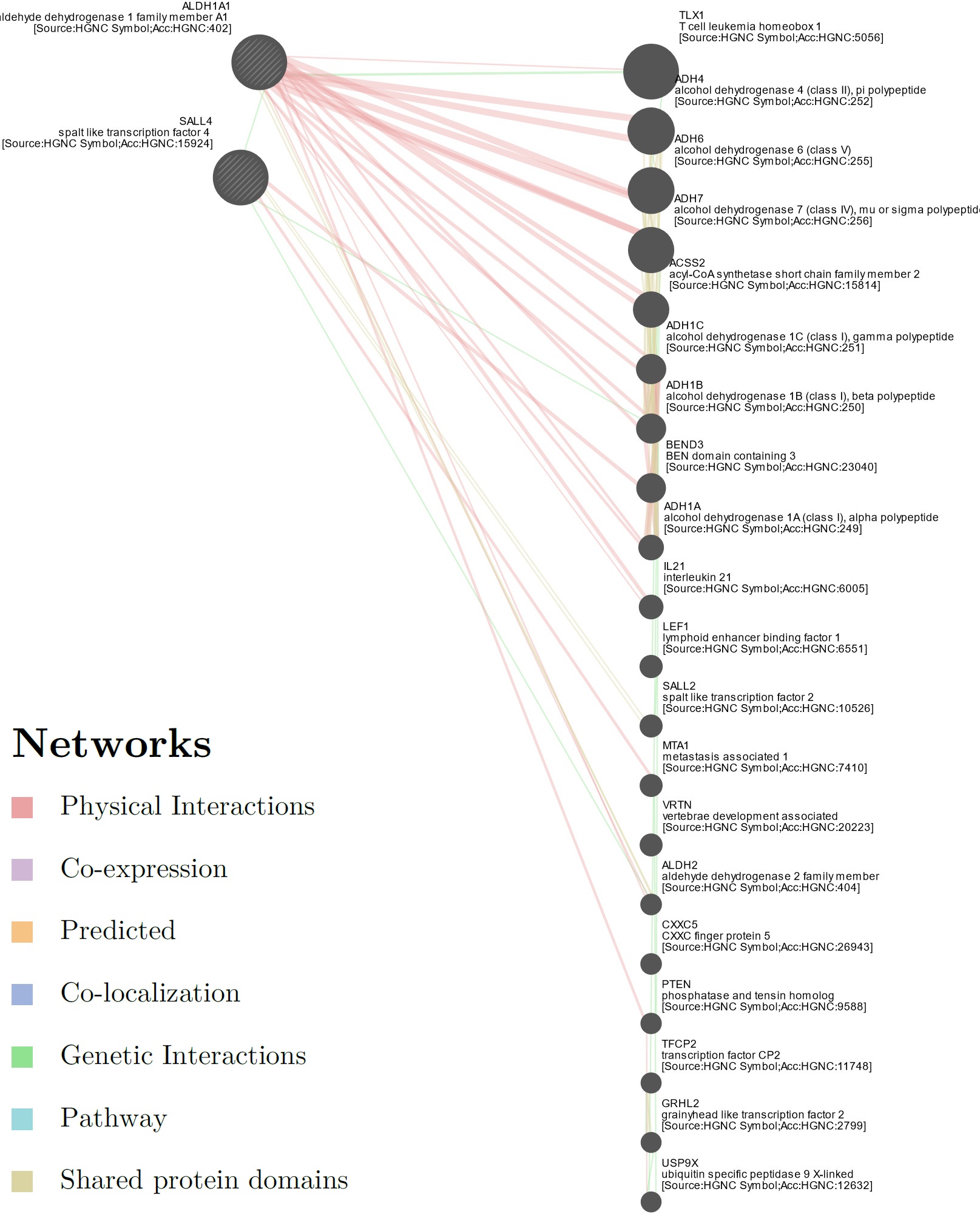

Supplement: Supplementary file 1 — Additional file 1: Supplementary Figure 1. Network analysis based on GeneMANIA prediction server for SALL4 and ALDH1A1. GeneMANIA analysis indicated gene sets that were enriched in the target network of SALL4 and ALDH1A1. Physical Interactions and Genetic are shown by distinct colors of the network edge for gene sets. [file 13048_2021_921_MOESM1_ESM.jpg]
